# Editorial: Insights in Elite Sports and Performance Enhancement: 2021

**DOI:** 10.3389/fspor.2022.960538

**Published:** 2022-06-22

**Authors:** Olivier Girard, Oliver Faude

**Affiliations:** ^1^School of Human Sciences (Exercise and Sport Science), The University of Western Australia, Perth, WA, Australia; ^2^Department of Sport, Exercise and Health, University of Basel, Basel, Switzerland

**Keywords:** high performance sport, effective communication, knowledge translation, coaches, data visualization for decision making

## Introduction

High performance sport is often perceived as the top end of athletic development, and typically operates in a fast paced, highly dynamic environment. In this environment, a large number of stakeholders is involved who pursue their own interests (sometimes with a commercial background) and exert influence on the athlete. This complex web of interests surrounds an individual, with his or her individual needs, requiring individual solutions to enable the athlete to achieve the small edge that is critical at the highest level. Despite its growth, the complex nature of managing high performance sport remains under-explored. The intention of this Research Topic is to shed more light on recent development in the elite sports and performance enhancement field. It is our hope that this articles collection will inspire, inform and provide direction and guidance to researchers/sport scientists in this area.

## ESSA Meet Ups

In April 2021, an *Exercise and Sports Science Australia* Meet Ups “Speaking coach—communicating sports science data for optimal impact” was held. During debates, it became clear that our effectiveness as researchers and/or sport scientists to inform practice hinges on our ability to communicate “*the right data at the right time in the right way*” to coaches and stakeholders. To this end, utilizing a range of strategies to support and narrate the message we wish to deliver become crucial.

Focussed discussions have resulted in the following key take home messages, that could be summarized as “*pitch at the audience you are speaking to*:”

- Be data informed and a good story teller
- Know when to present your data
- Use a shared language
- Simple done well far exceeds complex done poorly
- Data doesn't drive, it informs—numbers can be right but data can be misinterpreted
- Use peer-to-peer interaction for communication
- Coaches want clear, simple data that informs them of the risks and that they can act upon
- Be authentically interested to care
- Have a willingness to think, fail and reflect
- Be prepared to give and receive feedback
- Have the ability to hold critical conversations
- Be able to challenge but speak with empathy.

## This Issue

Using the example of winter sports to illustrate the importance of long-term athlete monitoring, Jordan et al. first presented a retrospective analysis of routine countermovement jump testing in female elite alpine skiers with and without anterior cruciate ligament reconstruction. Results highlight the relevance of including kinetic measures of vertical jump performance beyond vertical jump height, particularly, but not exclusively connected to the eccentric deceleration phase of the countermovement jump. These observations illustrate the importance of telling a story: know what message you wish to communicate to guide the selection of tools for the delivery method.

The second paper in this special issue continues on the topic of effective use of biomechanical data. Ozaki and Ueda determined the effects of clearing hurdles set at different heights (i.e., 10% higher and 10% lower than the center of mass) on hurdle clearance style and a range of kinematic data that may affect hurdle clearance performance. Original observations were that different hurdle heights alter not only spatiotemporal variables during hurdle clearance, but also the spatiotemporal variables associated with hurdle clearance velocity at each height. Another novel finding was that coach should consider that, even for the same hurdle height, spatiotemporal variables to focus on during training may differ depending on the height of the hurdler. In order to maximize “buy in,” it is important to match the level of complexity of the collected data and level of sports science understanding of end-users.

In the paper that follows, Seever et al. examined whether roller-massaging the calf muscles for three sets of 60 s per leg on 6 days a week over 2 weeks would improve ankle dorsiflexion range of motion and dynamic balance, as measured 1 and 7 days post-intervention. Authors reported increases in range of motion and dynamic balance that were still significant seven days post-intervention, emphasizing the sustainability of foam rolling effects. Practically, these findings support calf muscle roller-massaging for (i) warm-up when acute increases in ankle dorsiflexion range of motion are expected, and (ii) regular use to produce long-term improvements in ankle dorsiflexion range of motion and dynamic balance. Integrating this information in the practitioners' toolbox may facilitate implementation of foam rolling interventions.

The next paper illustrates that an apt description of physical training programs is essential for effective planning and delivery of performance enhancement and recovery strategies. In their analysis of 20 recently published papers on definition, concepts, benefits and the exercise employed in functional training programs, Ide et al. highlighted that functional training has no consistent and universal definition. Authors suggested that physical training programs should be described and classified as strength, power, flexibility, endurance, and the specific exercises selected. Such universal classification may in turn improve communication between athletes, coaches, sports scientists and researchers.

The final paper discusses how to balance data accuracy with interpretability, and improve decision making with very small samples and individual athletes by comparing Bayesian and frequentist analytical approaches in elite sports. As a proof-of-concept, Hecksteden et al. conducted a replicated cross-over trial on the effect of cold-water immersion on sprint performance recovery in soccer players. Bayesian analyses with informative priors (based on a published meta-analysis) may be a practicable option for data analysis, in particular when the analytical aim is on decision making rather than generalizable inference. Overall, maintaining the accuracy of the data that are communicated, whilst striving for ease of interpretation, is paramount for effective knowledge translation.

## Moving Forward

The papers within this Research Topic demonstrate that, in order for elite athletes to thrive, a disciplinary overlap becomes essential. The nature of discussions held during *Exercise & Sports Science Australia* Meet Ups would suggest that greater levels of acceptance of the delivered message may be achieved by telling a story using data that all stakeholders can relate to. This could be facilitated by understanding the environment the coaches and athletes operate in, including their challenges, pain points, “lingo” and terminology to deliver a message that lands. Ultimately, the new knowledge presented in this collection of articles should contribute to further our understanding of the complexity of elite sport.

## Author Contributions

Both authors listed have made a substantial, direct, and intellectual contribution to the work and approved it for publication.

## Conflict of Interest

The authors declare that the research was conducted in the absence of any commercial or financial relationships that could be construed as a potential conflict of interest.

## Publisher's Note

All claims expressed in this article are solely those of the authors and do not necessarily represent those of their affiliated organizations, or those of the publisher, the editors and the reviewers. Any product that may be evaluated in this article, or claim that may be made by its manufacturer, is not guaranteed or endorsed by the publisher.

